# Retraction: Stent Migration Following Endovascular Intervention in May-Thurner Syndrome

**DOI:** 10.7759/cureus.r63

**Published:** 2022-10-14

**Authors:** Dominick Steck, Suresh Keshavamurthy, Akshay Kumar, Sean Lyden, Eric Roselli

**Affiliations:** 1 Surgery, University of Washington School of Medicine, Seattle, USA; 2 Cardiothoracic Surgery, University of Kentucky, Lexington, USA; 3 Cardiothoracic Surgery, Cleveland Clinic Foundation, Cleveland, USA; 4 Vascular Surgery, Cleveland Clinic Foundation, Cleveland, USA

This article has been retracted as it has come to the attention of the journal that the article was submitted and published without the knowledge or consent of three of the listed authors (Dominick Steck, Sean Lyden, Eric Roselli), all of whom confirmed these allegations. Upon receipt of these allegations, the journal contacted authors Suresh Keshavamurthy and Akshay Kumar, and received the following reply from Dr. Keshavamurthy:

"This happened several years ago, and the initial draft was written by Dr. Steck after he observed the case. On the initial draft he had himself, me, Dr. Lyden and Dr. Roselli as potential co-authors in that order. However, due to personnel movement and procrastination this did not progress beyond the draft phase. A few months ago, while going through some old list of things to do, I discovered this draft and requested Dr.Kumar to take a look at it and see if he could put life into it. Dr.Kumar ran it with Dr. Roselli, worked on it and and was eventually able to submit it.

However, immediately on the day of submission he needed to travel to India for a family emergency and returned back couple weeks later. By this time the reviews were back, and I helped with the response to reviewers. Since Dr. Kumar thought he was facilitating with the submission process and improvements to the manuscript he was under the assumption everyone was on the same page. He agrees that we could have communicated better amongst ourselves and avoided the current situation.

Upon further reflection, venous stent technology has since improved and it was felt this (stent used in this case) did not represent the current iteration of stents. I wish to reiterate that the manuscript itself was a true representation of facts and there was an honest error in communication. While requesting retraction is neither easy nor desirable, for all the abovementioned reasons, we felt this was the best possible way to get closure."

